# Bilateral Non-Syndromic Supplemental Mandibular Incisors: Report on a Rare Clinical Case

**DOI:** 10.3390/children12101295

**Published:** 2025-09-25

**Authors:** Aldo Giancotti, Ilenia Cortese, Martina Carillo

**Affiliations:** Department of Systems Medicine, University of Rome “Tor Vergata”, Viale Oxford 81, 00133 Rome, Italy; giancotti@uniroma2.it (A.G.); ilenia.cortese20@gmail.com (I.C.)

**Keywords:** supplemental mandibular incisor, supernumerary tooth, non syndromic dental anomaly, tooth extraction, pediatric orthodontics

## Abstract

Background: Supplemental teeth are a rare subtype of supernumerary elements that closely resemble the morphology of normal dentition. Their occurrence in the mandibular anterior region is extremely uncommon. Aim: To describe the clinical features, diagnosis, and phased orthodontic management of a rare case involving bilateral supplemental mandibular incisors in a pediatric patient. Case report: A 7-year-old female patient presented with early mixed dentition and significant lower anterior crowding due to the presence of two fully erupted supplemental mandibular incisors. Treatment phase I included extraction of the malpositioned supplemental teeth and rapid maxillary expansion to transversally coordinate the arches. By the end of phase I, spontaneous alignment of the remaining lower incisors was observed. Discussion: The presence of two supplemental mandibular incisors is extremely rare in Caucasian populations. Supernumerary teeth can cause crowding, impaction, or delayed eruption of adjacent permanent teeth. Timely extraction can prevent such complications and often allows spontaneous alignment. Conclusions: The prompt removal of supplemental mandibular incisors, when they have just erupted, might lead to the alignment of the other incisors, considering that they spontaneously occupy the extractive spaces often without the aid of fixed appliances first line.

## 1. Introduction

Numeric dental anomalies include conditions in which the number of teeth exceeds the normal series. This condition is described as supernumerary teeth. When such teeth closely replicate the form and function of adjacent ones without anatomical deviations, they are classified as supplemental teeth. Conversely, those displaying atypical morphology are referred to as supernumerary teeth. This distinction aligns with the classification of numerical dental anomalies introduced by Tomes in 1873 [1].

The etiology of supernumerary and supplemental teeth has been attributed to atavistic traits, dental lamina hyperactivity, and dichotomy of the tooth germ theories. Among these, localized hyperactivity of the dental lamina is currently the most commonly accepted explanation for the formation of supernumerary elements [2,3,4,5,6,7,8].

Despite the supernumerary nature of teeth has been extensively explored, studies on supplemental teeth are rather sporadic in the literature.

As a matter of fact, the prevalence of supernumerary teeth has been thoroughly studied. Based on findings, single supernumerary teeth represent 76–86% of cases; double supernumerary teeth correspond to 12–23%; four supernumerary molars or distomolars account for 18% of cases. Finally, multiple supernumerary teeth represent less than 1% of all cases [9].

A study conducted by Jessica Mossaz et al. comprised a total of 101 supernumerary teeth. Most of the observed patients (80.5%) exhibited one single supernumerary tooth, while 15.8% had two supernumerary elements, and 3.7% had three. Males were affected more than females with a ratio of 1.65:1. Supernumerary teeth were most commonly conical in shape (42.6%), mostly with a normal or inclined vertical position (61.4%).

The aforementioned authors found that of the 19 supernumerary lateral incisors, 13 (68.4%) were located in the maxilla. The shape and position of the teeth were evenly distributed across the sample. Most of the supernumerary lateral incisors (18 out of 19; 94.7%) were located palatally or within the alveolar crest. The cusp of the supernumerary incisors was mostly located in the middle third of the root of the closest adjacent tooth (7 out of 19; 36.8%), and two lateral incisors (10.5%) had erupted [10].

Moreover, Hany M. Saber et al. evaluated the prevalence, distribution, characteristics, and complications of supernumerary teeth in the maxillary and mandibular anterior region in a sample consisting of Egyptian children. Most of their supernumerary teeth were single, vertically impacted in the palatal maxillary region, both conical and tuberculated in shape. More specifically, the number of supernumerary teeth per patient was single in 65% of the overall 189 patients observed, double in 31%, and triple in 4% of cases. The total number of supernumerary teeth was 248, including 247 in the maxilla (99.6%) and just one element in the mandible (0.4%) [11].

Based on the findings of Brook and Backman, the prevalence of supernumerary teeth in permanent dentition corresponds to approximately 1.5–3% in Caucasian populations, with a range between 80% and 94% of all supernumerary elements being identified in the premaxilla [12,13].

According to the literature, the presence of two supplemental mandibular incisors is rare among Asian and western populations. Indeed, the presence of six mandibular incisors has been reported to be extremely rare. Only two authors [14,15] described similar cases: Tanaka and Cho found bilateral completely erupted supernumerary teeth in the incisor region of the mandible. Both reported the characteristics of the single case observed and only Cho illustrated the extractive treatment of the two supernumerary teeth.

Hence, the herein article aims not only to report the characteristics of a case of a female pediatric patient with two supplemental mandibular bilaterally erupted permanent incisors, but also to share specific details on the treatment approach and outcomes. As a matter of fact, in the herein case, the extraction of supernumerary teeth led to a spontaneous alignment of lower anterior elements that exclusively required a close follow up by the clinician and, in turn, simplified the successive treatment phase II.

## 2. Case Report

**Figure 1:**
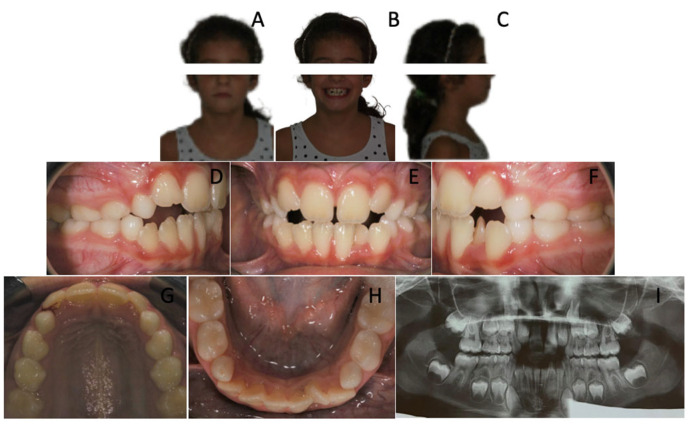
A 7-year-old female patient with two supplemental mandibular teeth. (**A**–**C**) Extra-oral photopraphs; (**D**–**H**) Intra-oral photographs; (**I**) Orthopantomography.

A 7-year-old female revealed early mixed dentition. Intraoral examination showed a symmetrical class II malocclusion (Figure 1) As a matter of fact, the upper and lower midlines were coincidental. Both the arches were constricted and the overbite was significantly decreased, resulting in an open bite tendency.

The lower arch showed significant anterior crowding. Indeed, the patient was noted to have six anterior mandibular teeth with fully developed clinical crowns: two central incisors, two lateral incisors, two supplemental teeth. Therefore, crowding was clearly due to the presence of two excess teeth.

The upper arch was slightly crowded and a lack of space for 1.2 and 2.2 was observed.

Phase I treatment objectives were

- lower crowding resolution;
- maxillary constriction correction.

**Figure 2:**
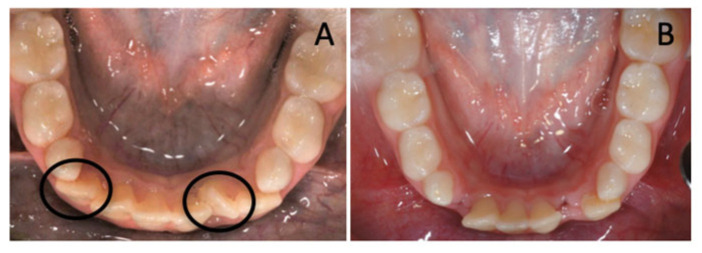
(**A**) Circle in black the supernumerary teeth selected for extractions. (**B**) The lower arch after the extractions.

The treatment plan comprised the extraction of supplemental teeth in order to correct the lower crowding and allow regular arch development. Considering the very similar morphology of the 6 incisors, the supplemental teeth that should have been extracted were selected according to their position. In the observed case, the incisor on the left side was extracted because it was the most rotated and farthest one from the arch perimeter. Therefore, it would have been unlikely for it to undergo a spontaneous realignment that would have more easily occurred for the other elements. Alongside, the other supernumerary tooth was selected for extraction, depending on its distal position on the right side that would have been an obstacle for the eruption of fourth quadrant dental elements (Figure 2).

**Figure 3:**
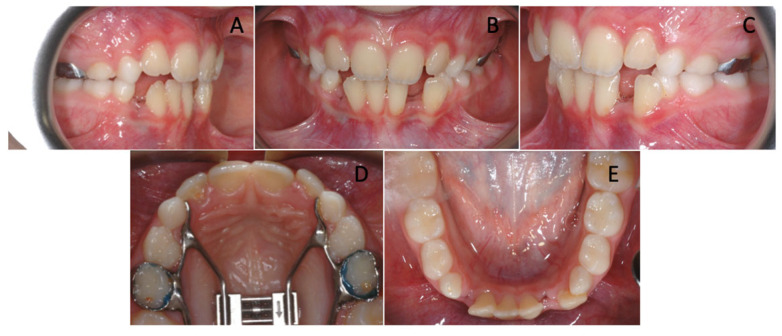
Extraction of the two supernumerary teeth and RME cementation in the upper arch. (**A**–**E**) Extraction of the supernumerary teeth in the lower arch and RME cementation in the upper ones.

In addition, a rapid maxillary expander was prepared on second deciduous molars to restore the correct transverse diameters and to gain more space for lateral incisors (Figure 3). After 6 initial activations, the appliance was activated twice a day for one week.

In the following months, the upper lateral incisors fully erupted, and the lower incisors improved their position exploiting the extractive spaces of the supernumerary teeth.

A grid plate was used as retention after maxillary expansion in order to control the open bite tendency and the tongue thrust that could affect the incisor position.

**Figure 4:**
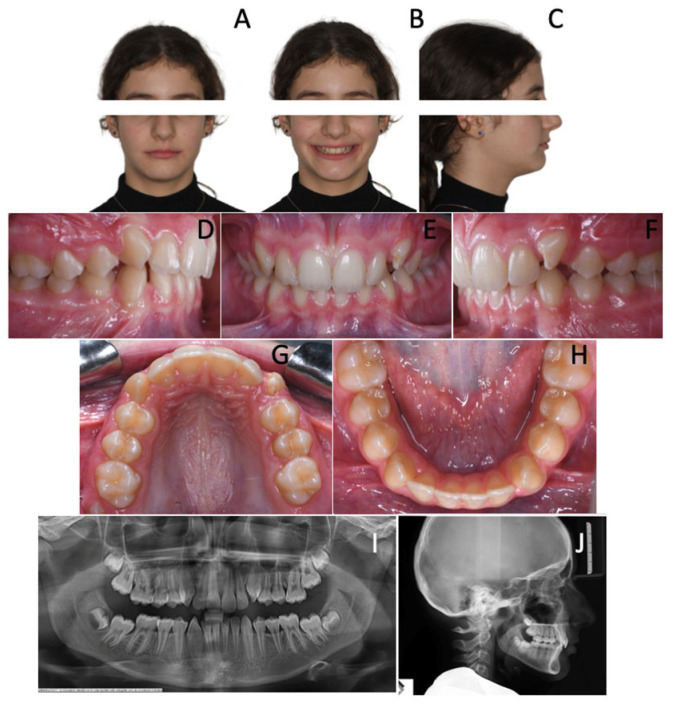
Patient at the end of phase I. (**A**–**C**) Extra-oral photographs; (**D**–**H**) Intra-oral photographs; (**I**) Orthopantomopraphy; (**J**) Teleradiography.

At the end of phase I (Figure 4), the overjet and overbite values were normalized, and, more importantly, the lower anterior crowding spontaneously resolved. Without the aid of fixed devices, lower incisors aligned, making the following step with a multibracket appliance significantly easier and more rapid (Figure 4).

As a matter of fact, in the second phase of treatment, the patient was treated with anterior bite raisers (BT2^®^, SIA Orthodontics Manufacturer Srl, Rocca d’Evandro, Italy) and a fixed appliance according to the bidimensional technique prescription [16].

Treatment objectives were the following:

- class II malocclusion correction to class I relationship;
- alignment, leveling and coordination of the arches;
- overjet and overbite correction;
- improvement of incisor torque;
- occlusion finishing;
- long-term stability.

**Figure 5:**
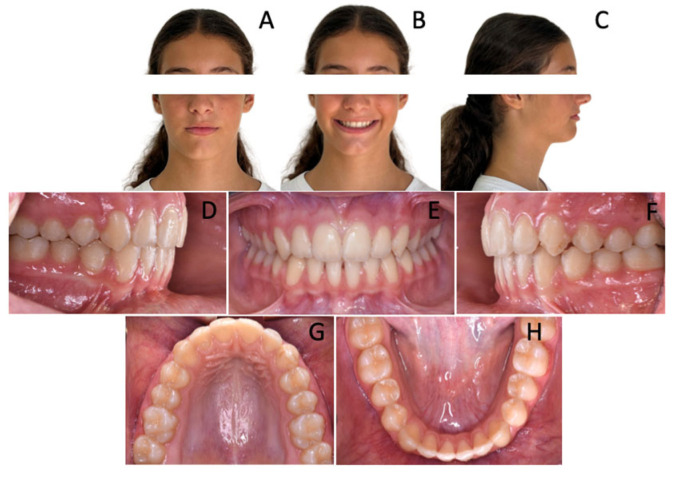
Patient at the end of the treatment. (**A**–**C**) Extra-oral photopraphs; (**D**–**H**) Intra-oral photopraphs.

**Figure 6:**
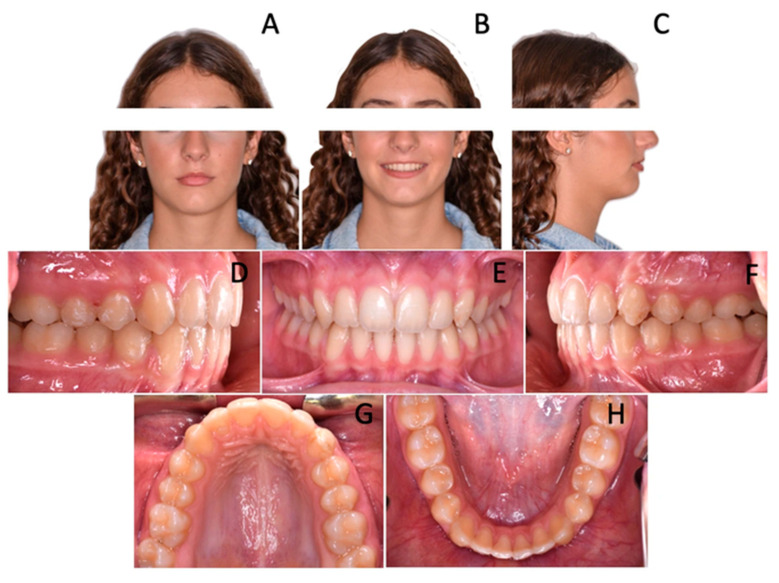
Follow up. (**A**–**C**) Extra-oral photographs; (**D**–**H**) Intra-oral photographs..

In 15 months of treatment, all the aforementioned objectives had been achieved, including final refinements that were performed in the final treatment phase featuring the use of multibracket appliances. By the end of treatment, a class I relationship was achieved on both sides, with alignment, leveling and coordination of the arches; overjet and overbite values were restored within normal ranges with adequate incisor torque. Moreover, proper occlusion was observed following the finishing phase (Figure 5). Finally, long-term stability was noted after 8 months from the conclusion of the treatment (Figure 6).

## 3. Discussion

Supernumerary teeth constitute a relatively uncommon developmental anomaly, especially when occurring in the mandibular arch and specifically in the incisor region, where their prevalence is extremely low.

The majority of reported cases involves a single supernumerary element, which is often incompletely developed or unerupted. Conversely, the presence of two well-formed supplemental teeth in the mandibular anterior region, as observed in the present case, is highly rare.

According to a comprehensive review of the literature, only two comparable cases have been documented to date, both occurring in Asian populations—specifically, one in Japan and one in China [14,15]. Across Caucasian individuals, the overall prevalence of single supplemental teeth ranges between 0.1% and 3.8%, while cases involving two supplemental teeth represent only a minor subset, with prevalence estimates ranging from 0.02% to 0.3% [12]. Therefore, such anomalies are not only rare but also clinically significant due to their potential interference with normal dental development and occlusion. Beyond the clinical implications, supplemental teeth serve as distinctive markers in forensic identification. Their low prevalence and high individuality confer a unique diagnostic value. Several studies have reported a strong positive correlation between supernumerary teeth and personal identification, supporting their use as distinctive dental markers in criminal investigations. Indeed, when standard identification methods are limited or unavailable, the presence of such anomalies can provide compelling evidence and serve as a powerful tool for human identification [17].

**Figure 7:**
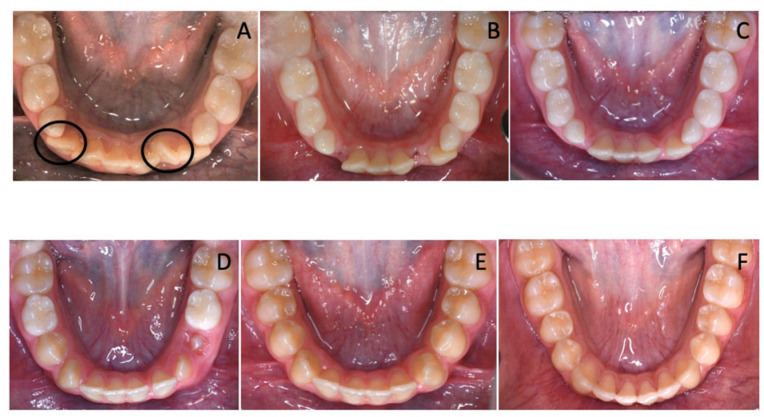
(**A**) The supplemental teeth selected for extractions circled in black; (**B**–**E**) Spontaneous lower incisor alignment after supplemental teeth extractions and (**F**) refinement of lower incisor alignment after multibracket therapy.

Clinically speaking, the early diagnosis of supplemental teeth is essential to optimize treatment outcomes and prevent complications. Supernumerary teeth may cause crowding, impaction, or delayed eruption of adjacent permanent teeth [18,19,20,21,22]. In many cases, timely extraction of the supernumerary teeth can help avoid these issues and restore dental alignment [20,23,24,25]. Spontaneous eruption of permanent teeth occurs in most of the cases in which supernumerary teeth have been removed [26,27]. However, if supernumerary teeth have already erupted, adjacent elements might spontaneously align after the extraction of supernumerary teeth, without the aid of orthodontic devices [28]. In these cases, when no additional orthodontic treatment is indicated, the ideal approach is to monitor the progress during dental exchange, thanks to the support of photographic records and documented clinical changes. In the case herein, the use of multibracket appliance or other devices during the first phase of treatment to promote the lower incisor alignment would have represented an overtreatment, given the fact that teeth can spontaneously move towards extractive spaces [29,30,31,32,33] (Figure 7A–E). As a matter of fact, the second phase of treatment with multibracket appliance required a very slight refinement of the alignment already achieved thanks to physiological repositioning of the mandibular incisors in the space gained (Figure 7F).

Moreover, the selection of the supernumerary cases can be very simple in cases where they are microdontic or otherwise very different in morphology from their permanent counterparts, as was the case illustrated by Tanaka [14]. On the contrary, distinguishing between a normal tooth and its supplemental counterpart might be challenging when both are similarly well-developed, as happened in the case described by Cho [15] and in the present case. In such scenarios, the decision to extract depends on position, prioritizing the extraction of the more malpositioned tooth to prevent or alleviate crowding [23].

In the herein case, thanks to a careful clinical decision-making process, the extraction of the two supplemental mandibular incisors alone was sufficient to correct the space deficiency in the lower arch, allowing for the spontaneous realignment of the remaining teeth. This underscores the potential benefit of conservative intervention when anomalies are early diagnosed and appropriately managed.

## 4. Conclusions

Early diagnosis of mandibular supplemental teeth plays a decisive role in preventing crowding, impaction, or eruptive anomalies that may compromise regular dental development. When such teeth are already erupted, the selective extraction of the most malpositioned elements can lead to the spontaneous realignment of the anterior segment. In these situations, orthodontic alignment of the unextracted elements would represent an overtreatment on behalf of the clinician, who should, instead, opt for a minimally invasive approach based on careful clinical monitoring, supported by photographic records. A conservative and timely strategy not only simplifies subsequent orthodontic phases, but also improves treatment efficiency and prognosis.

Beyond its clinical relevance, the presence of two supplemental teeth in the mandibular anterior region represents an extremely rare dental anomaly, particularly across the Caucasian population. This condition’s rare incidence calls for detailed reporting and a systematic analysis of similar cases. The herein case contributes to extending the existing literature by documenting a successful treatment outcome achieved by means of the extraction of supplemental teeth alone. This result underscores the importance of promptly recognizing and suitably managing such anomalies, while highlighting the potential benefits of a minimally invasive approach and the importance of careful clinical decision-making.

Looking to the future, it would be interesting to report similar clinical cases focusing on longer-term follow-up and stability.

## Data Availability

The original contributions presented in the study are included in the article, further inquiries can be directed to the corresponding author.
